# Immunomodulatory Properties of Polysaccharides from the Coral *Pseudopterogorgia americana* in Macrophages

**DOI:** 10.3390/cells10123531

**Published:** 2021-12-14

**Authors:** Oleg V. Chernikov, Hsiao-Wen Chiu, Lan-Hui Li, Maxim S. Kokoulin, Valentina I. Molchanova, Hsien-Ta Hsu, Chen-Lung Ho, Kuo-Feng Hua

**Affiliations:** 1 G.B. Elyakov Pacific Institute of Bioorganic Chemistry FEB RAS, 690022 Vladivostok, Russia; chernikovoleg@gmail.com (O.V.C.); maxchem@mail.ru (M.S.K.); molchanova_val@mail.ru (V.I.M.); 2 Department of Biotechnology and Animal Science, National Ilan University, Ilan 260007, Taiwan; looking123123@hotmail.com; 3 Department of Laboratory Medicine, Linsen, Chinese Medicine and Kunming Branch, Taipei City Hospital, Taipei 108, Taiwan; A1525@tpech.gov.tw; 4 Department of Pathology, Tri-Service General Hospital, National Defense Medical Center, Taipei 11490, Taiwan; 5 Division of Neurosurgery, Taipei Tzu Chi Hospital, Buddhist Tzu Chi Medical Foundation, New Taipei City 231, Taiwan; j1208192@ms45.hinet.net; 6 School of Medicine, Buddhist Tzu Chi University, Hualien 970, Taiwan; 7 Division of Wood Cellulose, Taiwan Forestry Research Institute, Taipei 100051, Taiwan; hochenlung@gmail.com; 8 Department of Medical Research, China Medical University Hospital, China Medical University, Taichung 404333, Taiwan

**Keywords:** *Pseudopterogorgia americana*, polysaccharide, macrophages, immune modulation

## Abstract

Polysaccharides from marine organisms produce an important regulatory effect on the mammalian immune system. In this study, the immunomodulatory properties of a polysaccharide that was isolated from the coral *Pseudopterogorgia americana* (PPA) were investigated. PPA increased the expression levels of tumour necrosis factor-α (TNF-α), interleukin-6 (IL-6) and cyclooxygenase-2 (COX-2), but not inducible nitric oxide synthase and nitric oxide, in macrophages. A mechanistic study revealed that PPA activated macrophages through the toll-like receptor-4 and induced the generation of reactive oxygen species (ROS), increased the phosphorylation levels of protein kinase C (PKC)-α, PKC-δ and mitogen-activated protein kinases (MAPK), and activated NF-κB. The inhibition of ROS and knockdown of PKC-α reduced PPA-mediated TNF-α and IL-6 expression; however, the knockdown of PKC-δ significantly increased PPA-mediated TNF-α expression. In addition, the inhibition of c-Jun N-terminal kinase-1/2 and NF-κB reduced PPA-mediated TNF-α, IL-6 and COX-2 expression. Furthermore, the inhibition of ROS, MAPK and PKC-α/δ reduced PPA-mediated NF-κB activation, indicating that ROS, MAPK and PKC-α/δ function as upstream signals of NF-κB. Finally, PPA treatment decreased the phagocytosis activity of macrophages and reduced cytokine expression in bacteria-infected macrophages. Taken together, our current findings suggest that PPA can potentially play a role in the development of immune modulators in the future.

## 1. Introduction

The ocean is rich in biodiversity and this makes it a very important source for biomedical researchers to develop active substances. The interest is due, first, to the ocean’s high content of biologically active substances, which are effective in the treatment and prevention of multiple diseases. In addition, they are used in the food, cosmetic and other industries, and thus biologically active substances must be extracted from these organisms [1]. Thanks to systematic studies of marine invertebrates (bivalve molluscs, sponges, echinoderms, etc.), multidimensional data have been obtained confirming that marine invertebrates are an important source of polysaccharides or glycoconjugates, which are noncovalently bound complexes comprising a polysaccharide with a protein component. Polysaccharides have attracted special attention because they exhibit a range of biological properties without toxicity [2]. Depending on many factors, such as the chemical structure of the polysaccharides, the route of administration, and the type of the disease, polysaccharides affect immune cells, such as phagocytes, T cells and the complement system, and consequently affect cellular and humoural immunity [3,4].

Marine-derived polysaccharides are an important resource for the development of immunomodulators. Sulfated polysaccharides from sea cucumbers, fucoidan from brown algae and iota-carrageenan from red algae showed inhibitory activity against SARS-CoV-2 to combat COVID-19 [5,6,7]. Sulfated polysaccharides from *Codium fragile* (green sea fingers) enhance the cytotoxicity of natural killer cells toward HeLa cells by increasing the release of cytokines and cytotoxic granules [8]. Polysaccharides from *Chlamys farreri* (marine bivalve mollusc) and sea cucumber viscera promote phagocytosis and increase cytokine production in macrophages [9,10]. The water-soluble polysaccharides from *Cuminum cyminum* activate RAW264.7 cells and NK-92 cells, causing them to produce cytokines [11]. Marine-derived polysaccharides also possess various useful physicochemical and biological properties, including antioxidant activities [12], antitumour activities [13] and prebiotic functions [14]. In addition, marine-derived polysaccharides have medicinal potential in the treatment and prevention of disorders, such as diabetes [15], renal disease [16], cardiovascular disease [17] and neurodegenerative disease [18]. However, limited information is available on the isolation and study of high molecular weight carbohydrates from corals. In the latter part of the last century, numerous studies were conducted to dissect the chemical composition of coral mucus. The major secretory product of hard (Scleractinia) and soft (Alcyonacea) corals is mucus, which consists of varying proportions of proteins, lipids and polysaccharides [19,20]. The chemical composition of the mucus glycoprotein differs among coral species [21].

**Figure 1:**
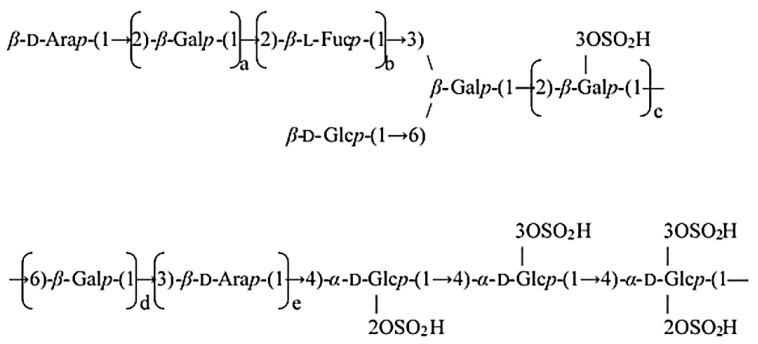
Proposed chemical structure of PPA.

We previously isolated mucus glycoprotein from the soft coral *Pseudopterogorgia americana*, which is widespread in the waters of the Caribbean Sea, and its physicochemical and biological properties were studied [22,23]. The carbohydrate part of the glycoprotein is a sulfated glucuronoglycan named PPA. The polysaccharide component was shown to represent a sulfated glucuronoglycan because D-glucuronic acids (9%) and sulfate groups (more than 6%) were detected. We confirmed that PPA contains sulfate groups because we detected an absorption band at 840 cm^−1^ in the IR spectrum. In addition, the residues of D-arabinose, D-fucose, D- and L-galactose, and D-glucose in molar ratios of 35.6:24.5:33.3:6.6 were identified in a hydrolysate of PPA. The chemical structure of PPA was characterized by methylation studies, partial acid hydrolysis, periodate oxidation and Smith degradation. A study of oligosaccharides obtained by partial acid hydrolysis of PPA showed that the polysaccharide is branched; linear sections of the carbohydrate chain with 1,2-bonds between the galactose residues and 1,4-bonds between the glucose residues are present. Residues of D-arabinose and D-fucose occupy the terminal position and are incorporated in the carbohydrate chain by 1,3- and 1,2-bonds, respectively. Sulfate groups are located at C-2 and C-3 of D-glucose residues and at C-3 of L-fucose residues [23]. The structural features were obtained as shown in Figure 1.

Macrophages are one of the important antigen-presenting cells that play important roles in innate immunity and regulate adaptive immunity. Cytokines secreted from activated macrophages cause inflammatory responses and are essential for host defences against invading pathogens [24]. However, uncontrolled and prolonged inflammatory responses are harmful to the host and promote the pathogenesis of many inflammatory diseases, such as metabolic disorders [25]. It is of great research significance to develop polysaccharides with immunomodulatory functions and understand their structure and mechanism of action. In this study, the immunomodulatory properties of a polysaccharide isolated from the coral *Pseudopterogorgia americana* (PPA) were investigated. The cytokine induction activity of PPA was investigated and the signalling pathways that regulate PPA-mediated cytokine production were also dissected. The effect of PPA on the phagocytic activity of macrophages in response to bacterial infection was also studied.

## 2. Materials and Methods

### 2.1. Reagents and Chemicals

Antibodies against proIL-1β, iNOS, COX-2, NF-κB SN50 cell permeable inhibitory peptide (sc-3060) and pyrrolidine dithiocarbamic acid ammonium salt (PDTC) were obtained from Santa Cruz Biotechnology (Santa Cruz, CA, USA). Antibodies against actin, LPS, pharmaceutical inhibitors, N-acetyl cysteine (NAC), polymyxin B (PMB) and dimethyl sulfoxide (DMSO) were obtained from Sigma-Aldrich (St. Louis, MO, USA). PD98059, SB203580, SP600125 and antibodies against phospho-proteins were obtained from Cell Signaling Technology (Beverly, MA, USA). The NF-κB reporter reagent (QUANTI-Blue) and ATP were obtained from InvivoGen (San Diego, CA, USA). ELISA kits were obtained from Affymetrix eBioscience (San Diego, CA, USA). The intracellular ROS indicator was obtained from Molecular Probes (Eugene, OR, USA). The florescent E. coli particles and Pierce Limulus Amebocyte Lysate (LAL) Chromogenic Endotoxin Quantitation Kit were obtained from Thermo Fisher Scientific (Waltham, MA, USA). M-CSF was obtained from Peprotech (London, UK). Lipid IVa was obtained from MyBioSource (San Diego, CA, USA).

### 2.2. Cell Cultures

J774A.1 macrophages, RAW264.7 macrophages and THP-1 monocytes were obtained from the American Type Culture Collection (Rockville, MD, USA). THP-1 monocytes were incubated for 48 h with 50 nM PMA and differentiated THP-1 macrophages were obtained by washing out the nonadherent cells. Stable PKC-α and PKC-δ knockdown RAW264.7 and mock cells were obtained from stable transfection with shRNA plasmids that target PKC-α (catalogue number: TG501653), PKC-δ (catalogue number: TR513052) and a scrambled sequence (catalogue number: TR30012), respectively, which were obtained from OriGene Technologies, Inc. (Rockville, MD, USA). The RAW-Blue cell line is a RAW264.7 cell-based NF-κB reporter cell line that was obtained from InvivoGen (San Diego, CA, USA). Primary wild-type and TLR4-knockout macrophages were obtained from the bone marrow of wild-type and toll-like receptor 4 (TLR4)-knockout mice, respectively. Briefly, bone marrow cells were isolated from C57BL/6 mice femur and tibia and incubated for 7 days in a culture medium containing M-CSF. The isolation of bone marrow from mice was performed with the approval of the Institutional Animal Care and Use Committee of the National Ilan University (approval number: No. 102-40).

### 2.3. Isolation of PPA

PPA was isolated, and its structure was established as described in previous reports [22,23]. Briefly, 1 kg of the fresh soft coral *Pseudopterogorgia americana* was ground into small pieces, homogenized with a 0.9% solution of sodium chloride in a ratio of 1:0.5 at 4–6 °C for 15 min, and added to a 0.9% solution of sodium chloride to 5 L for extraction (starting material:extractant = 1:5, *w*/*v*) with stirring for 12–14 h at 4–6 °C. Then, the mixture was centrifuged for 30 min at 4000 rpm, and the insoluble material was discarded. A four-fold volume of 96% ethanol (supernatant:ethyl alcohol = 1:4) cooled to 4 °C was added to the supernatant to precipitate the high molecular weight fraction. For more complete precipitation, the mixture was incubated in a refrigerator for 12–14 h at 4 °C. As a result, a high molecular weight fraction in the form of flocculent flakes floating on the surface of the solvent was obtained. After filtration through a nylon membrane, the precipitate was wrung out, washed three to four times with anhydrous 96% ethyl alcohol that had been cooled to 4 °C, dried in air at room temperature (19–20 °C) and dissolved in distilled water until complete dissolution (requiring approximately 3 L of distilled water) with magnetic stirring. The resulting viscous solution was dialyzed against distilled water for 70–72 h with a double change of water daily. The dialyzed product was freeze-dried, and a snow-white powder with a yield of 19.0 g (1.9% of the raw material weight) was obtained. The homogeneity of the PPA was confirmed according to gel filtration. The gel filtration chromatography showed that the PPA formed a single symmetrical peak, indicating homogeneity of preparation. The glucose content in the PPA is around 55.3%, and determined by the phenol-sulfuric method, using glucose as standard. The protein and nucleic acid content in the PPA was less than 2% and 0.8% respectively [23]. The LPS concentration in the PPA was analysed using an LAL test following the manufacturer’s instructions, and the LPS concentration was below 1 endotoxin unit/mL.

### 2.4. Activation of Macrophages by PPA and Bacteria

For IL-1β precursor (proIL-1β) production, J774A.1 macrophages were incubated with PPA or LPS (1 µg/mL) for 6 h with or without PMB (10 µg/mL). The level of proIL-1β in the cell lysates was analysed using Western blotting. For IL-1β production, J774A.1 macrophages were incubated with PPA or LPS (1 µg/mL) for 5 h, followed by stimulation with ATP (5 mM) for 0.5 h. The levels of IL-1β in the supernatants were analysed using an ELISA. For COX-2, IL-6, TNF-α, iNOS and NO production, RAW264.7 macrophages or THP-1 macrophages were incubated with PPA for 24 h. The concentration of cytokines and NO in the supernatants was analysed using ELISAs and the Griess reaction, respectively. The levels of COX-2, iNOS and actin in the cell lysates were analysed using Western blotting. For the phosphorylation levels of MAPKs, PKC-α/δ and IκB-α, RAW264.7 macrophages were incubated with 10 µg/mL PPA for 0–60 min, and protein phosphorylation levels were analysed using Western blotting. For the effect of MAPK inhibitors on the phosphorylation levels of MAPK in PPA-stimulated cells, the cells were treated with 10 µg/mL PPA for 20 min. All the inhibitors were added to culture medium 0.5 h before PPA or LPS treatment. For bacteria-induced cytokine production, J774A.1 macrophages were incubated with 10 µg/mL PPA, 0.1 µg/mL LPS or sterile H_2_O (5 µL/mL) for 24 h, followed by changing to a fresh medium. The cells were infected with *Shigella sonnei* at a multiplicity of infection (MOI) of 50 or *Escherichia coli* at an MOI of 30 for 5 h. The levels of TNF-α and IL-6 in the supernatants were analysed using an ELISA.

### 2.5. Detection of Cytokines

Briefly, we added 50 µL of each sample or standard to the antibody-coated 96-well plate, followed by adding 50 µL of biotin conjugate (detection antibody) to the wells. The plate was covered with an adhesive film and incubated at room temperature for 2 h on a microplate shaker. After washing the plate three times with washing buffer (PBS with 0.05% Twee 20), 100 µL of diluted Streptavidin-HRP was added to all wells and incubated at room temperature for 1 h on a microplate shaker. After washing, 100 µL of TMB substrate solution was added to all wells and the plate was incubated for 30 min in the dark. A total of 100 µL of stop solution (1 M phosphoric acid) was added to all wells when the highest standard developed a dark blue colour. The absorbance of the wells was measured using an ELISA microplate reader with 450 nm as the primary wavelength and 620 nm as the reference wavelength.

### 2.6. Detection of Cytokines and NO

Briefly, we added 50 µL of each sample or standard to the 96-well plate, followed by adding 50 µL of sulfanilamide solution (1% sulfanilamide in 5% phosphoric acid) to the wells. The plate was incubated at room temperature for 10 min in the dark, followed by adding 50 µL of NED solution (0.1% N-1-napthylethylenediamine dihydrochloride in water) to all wells and being incubated at room temperature for an additional 10 min in the dark. The absorbance of the wells was measured using an ELISA microplate reader at 540 nm.

### 2.7. Analysis of NF-κB Activity

RAW-Blue cells, an NF-κB reporter cell line, are derived from RAW264.7 macrophages, which stably express a secreted embryonic alkaline phosphatase (SEAP) gene that is inducible by an NF-κB transcription factor. RAW-Blue cells were treated with PDTC, sc-3060 or DMSO (vehicle) (1 µL/mL) for 0.5 h and then stimulated with PPA or sterile H_2_O (vehicle) (5 µL/mL) for 24 h. Twenty microliters of conditioned medium were mixed with 180 μL of SEAP detection medium QUANTI-Blue for 30 min in a 96-well plate. The NF-κB activity was analysed by measuring the SEAP levels using an ELISA microplate reader at 620 nm.

### 2.8. Detection of ROS Generation

RAW264.7 macrophages were treated with NAC or sterile H_2_O (vehicle) (5 µL/mL) for 0.5 h and then with 2 µM intracellular indicator (2′,7′-dichlorofluorescein diacetate) for 0.5 h. Cells were stimulated with 10 μg/mL PPA or 1 μg/mL LPS for 0–60 min. The ROS production was analysed by measuring the fluorescent signal using a fluorescent photometer.

### 2.9. Phagocytosis Assay

For the analysis of bacterial phagocytosis with the J774A.1 macrophages, cells were treated with 10 µg/mL PPA, 0.1 µg/mL LPS or sterile H_2_O (vehicle) (5 µL/mL) for 24 h. Then, the cells were infected with *S. sonnei* at an MOI of 50 or *E. coli* at an MOI of 30 for 15 min, and the extracellular bacteria were washed out by PBS. To make sure that there was no extracellular bacterial growth, cells were incubated for 1 h at 4 °C with PBS containing 10 µg/mL gentamicin, an antibiotic that has no effect on intracellular bacterial growth. The intracellular bacterial growth was analysed by counting the colony-forming units (CFUs). For the analysis of the phagocytosis of fluorescent beads with the J774A.1 macrophages, cells were treated with 10 µg/mL PPA, 0.1 µg/mL LPS or sterile H_2_O (vehicle) (5 µL/mL) for 24 h. Then, the cells were treated for 1 h with 2 µg/mL pHrodo Green *E. coli* BioParticles Conjugate. The fluorescent signal was analysed using flow cytometry.

### 2.10. Analysis of mRNA Levels of PKC

Total RNA was isolated from PKC-α knockdown, PKC-δ knockdown or control cells, and the cDNA were synthesized using reverse transcription. Quantitative PCR was performed with a StepOne real-time PCR system purchased from Applied Biosystems (Foster City, CA, USA). The mRNA levels were normalized to GAPDH. The primers were purchased from Genomics (Taipei, Taiwan), and the sequences are as follows: PKC-α, forward: 5′-ttcccaatcatcatagcaca-3′; PKC-α, reverse: 5′-gagatagttatcaaccgagcag-3′; PKC-δ, forward: 5′-gttcatcgccaccttctttg-3′; PKC-δ, reverse: 5′-atttcttatggatggcagcg-3′. GAPDH, forward: 5′-gaagggtggagccaaaagg-3′; GAPDH, reverse: 5′-gatggcatggactgtggtca-3′.

### 2.11. Statistical Analysis

Two-tailed *t*-tests and ANOVA with Dunnett’s multiple comparisons test were used for the statistical analysis of two groups and three or more groups, respectively. Error bars represent standard deviations of three separate experiments. *, ** and *** represent *p* < 0.05, *p* < 0.01 and *p* < 0.001, respectively.

## 3. Results

### 3.1. PPA Activates Macrophages Independent of LPS Contamination

**Figure 2:**
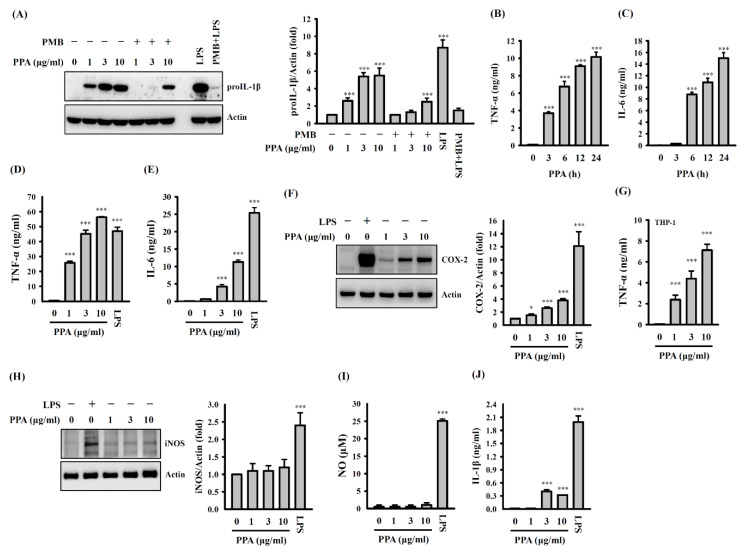
Effect of PPA on inflammatory mediator expression. (**A**) Effect of PMB on proIL-1β expression in PPA- or LPS-stimulated J774A.1 macrophages. (**B**) TNF-α expression in PPA-stimulated J774A.1 macrophages. (**C**) IL-6 expression in PPA-stimulated J774A.1 macrophages. (**D**) TNF-α expression in PPA- or LPS-stimulated RAW264.7 macrophages. (**E**) IL-6 expression in PPA- or LPS-stimulated RAW264.7 macrophages. (**F**) COX-2 expression in PPA- or LPS-stimulated RAW264.7 macrophages. (**G**) TNF-α expression in PPA-stimulated THP-1 macrophages. (**H**) iNOS expression in PPA- or LPS-stimulated RAW264.7 macrophages. (**I**) NO expression in PPA- or LPS-stimulated RAW264.7 macrophages. (**J**) IL-1β expression in PPA- or LPS-stimulated RAW264.7 macrophages. * *p* < 0.05 and *** *p* < 0.001 compared to control cells.

The structural features of PPA are shown in Figure 1 [23]. IL-1β is one of the key signalling molecules for both innate and adaptive immunity. IL-1β is produced as an IL-1β precursor (proIL-1β) by the activated macrophages and its cleavage is caused by caspase-1, which is regulated by the inflammasomes [26]. The effect of PPA on proIL-1β expression was investigated to determine whether PPA activates macrophages. We found that proIL-1β expression was induced in cells treated with 1, 3 and 10 μg/mL of PPA (*p* < 0.001) (Figure 2A). We tested the effect of positively charged polymyxin B (PMB) (10 µg/mL), a LPS inhibitor, on PPA-mediated proIL-1β expression to check the possible contamination of the LPS in the PPA. The proIL-1β expression in the PPA-stimulated cells was reduced by PMB, while proIL-1β expression in the LPS-stimulated cells was completely inhibited by PMB (Figure 2A). PPA is a negatively charged, sulfated polysaccharide, which may be affected by positively charged PMB. In addition, 10 µg/mL of PPA time-dependently increased the levels of TNF-α (*p* < 0.001) (Figure 2B) and IL-6 expression (*p* < 0.001 at 6–24 h) (Figure 2C). PPA dose-dependently increased the levels of TNF-α (*p* < 0.001) (Figure 2D), IL-6 (*p* < 0.001) (Figure 2E) and COX-2, the inducible form of cyclooxygenase that catalyses the conversion of arachidonic acid to prostaglandins, which played a pivotal role in the inflammatory responses [27] (*p* < 0.05 at 1 µg/mL; *p* < 0.001 at 3 and 10 µg/mL) (Figure 2F). Furthermore, PPA dose-dependently induced TNF-α expression in human THP-1 macrophages (*p* < 0.001), confirming the macrophage-activation property of PPA (Figure 2G). Notably, LPS significantly increased the levels of inducible nitric oxide synthase (iNOS) (*p* < 0.001) (Figure 2H) and nitric oxide (NO) (*p* < 0.001) (Figure 2I) in RAW264.7 macrophages, while PPA did not. LPS and ATP also significantly increased IL-1β expression in J774A.1 macrophages (*p* < 0.001); however, the IL-1β expression that was increased by PPA and ATP was less than that of LPS and ATP (*p* < 0.001 at 3 and 10 µg/mL) (Figure 2J). These results confirmed that PPA activated macrophages independently of LPS contamination.

### 3.2. PPA Activates Macrophages through TLR4

**Figure 3:**
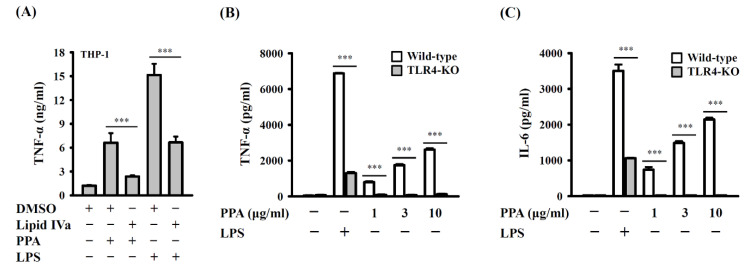
PPA activates macrophages through TLR4. (**A**) Effect of lipid IVa on TNF-α expression in PPA- or LPS-stimulated THP-1 macrophages. (**B**) TNF-α expression in PPA- or LPS-stimulated wild-type or TLR4 knockout BMDM. (**C**) IL-6 expression in PPA- or LPS-stimulated wild-type or TLR4 knockout BMDM. *** *p* < 0.001.

To investigate whether PPA activates macrophages through TLR4, THP-1 macrophages were incubated with 1 μg/mL of lipid IVa, a competition TLR4-MD2 inhibitor, for 0.5 h before 10 μg/mL of PPA or 1 μg/mL of LPS treatment for 24 h. We found that lipid IVa significantly reduced TNF-α expression in PPA- or LPS-activated THP-1 macrophages (*p* < 0.001) (Figure 3A). To further confirm the role of TLR4 in PPA-mediated cytokine production, bone marrow-derived macrophages (BMDM) from wild-type and TLR4 knockout mice were incubated with PPA or 1 μg/mL of LPS for 6 h. We found that PPA and LPS significantly increased the expression levels of TNF-α and IL-6 in wild-type BMDM; however, the expression levels of TNF-α and IL-6 in TLR4 knockout BMDM were impaired (*p* < 0.001) (Figure 3B,C). These results indicated that TLR4 was a putative receptor for PPA.

### 3.3. PPA-Induced TNF-A and IL-6 Expression through Reactive Oxygen Species (ROS) and Protein Kinase C (PKC)-A

**Figure 4:**
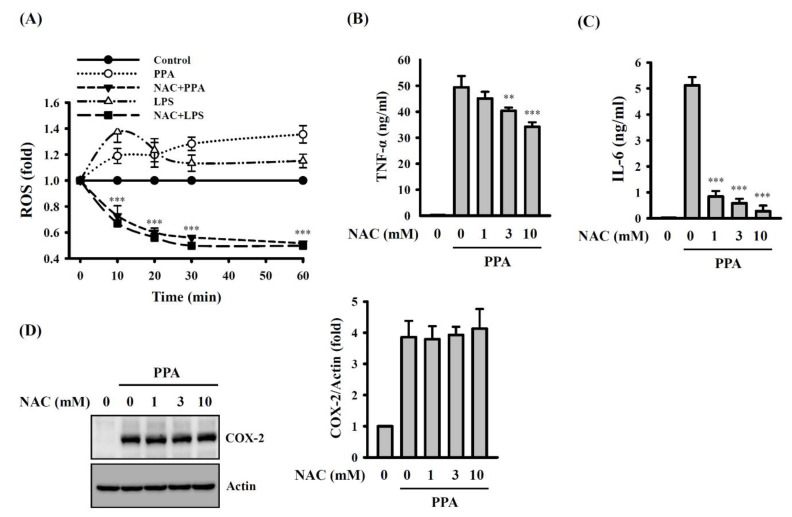
PPA induces TNF-α and IL-6 secretion through ROS in RAW264.7 macrophages. (**A**) ROS production in PPA- or LPS-stimulated cells. (**B**–**D**) Effect of NAC on TNF-α (**B**), IL-6 (**C**) and COX-2 (**D**) in PPA-stimulated cells. ** *p* < 0.01 and *** *p* < 0.001 compared to PPA-treated cells.

Important signalling pathways were investigated to dissect the cellular mechanisms that regulate PPA-mediated macrophage activation. It has been demonstrated that cytokine expression is positively regulated by the ROS in macrophages [28]. ROS production in RAW264.7 macrophages was significantly increased by 10 μg/mL of PPA or 1 μg/mL of LPS, and these effects were reduced by 10 mM N-acetyl-cysteine (NAC), a ROS scavenger (*p* < 0.001) (Figure 4A). In addition, NAC significantly inhibited the expression levels of TNF-α (*p* < 0.01 at 3 mM; *p* < 0.001 at 10 mM) (Figure 4B) and IL-6 (*p* < 0.001) (Figure 4C) in PPA-activated macrophages. However, NAC did not affect PPA-induced COX-2 expression (Figure 4D). Based on these results, ROS positively regulated TNF-α and IL-6 secretion in the PPA-treated cells.

**Figure 5:**
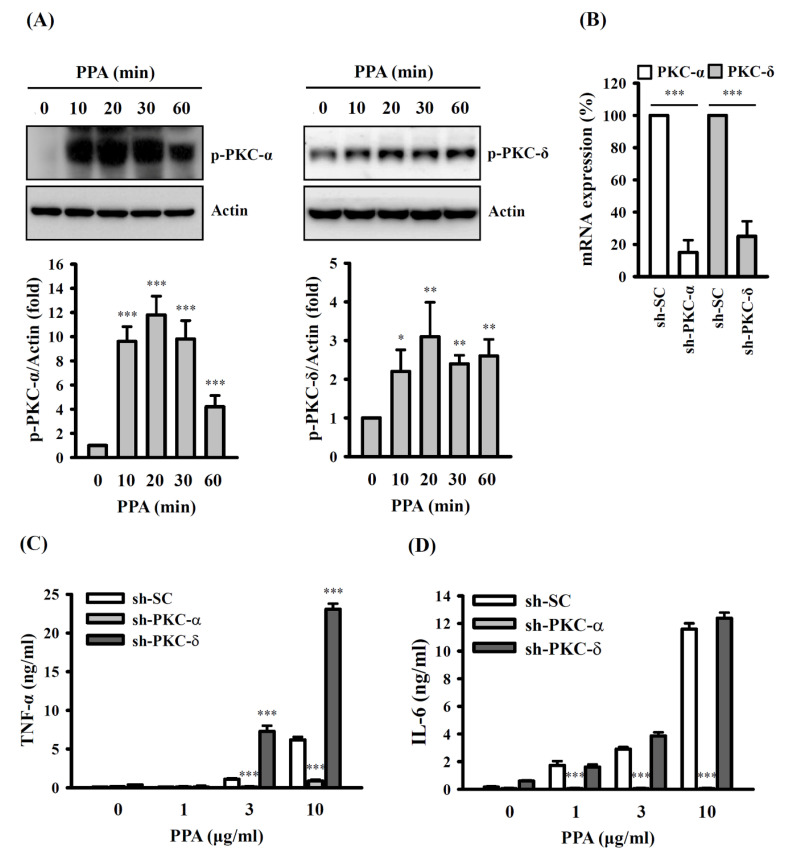
Effect of PKC-α/δ on PPA-mediated TNF-α and IL-6 expression in RAW264.7 macrophages. (**A**) The phosphorylation levels of PKC-α and PKC-δ in PPA-stimulated cells. (**B**) The mRNA expression of sh-PKC-α, sh-PKC-δ and sh-SC cells. (**C**) TNF-α and (**D**) IL-6 expression in PPA-stimulated sh-PKC-α, sh-PKC-δ and sh-SC cells. * *p* < 0.05, ** *p* < 0.01 and *** *p* < 0.001 compared to control cells (**A**), sh-SC cells (**C**,**D**) or as indicated (**B**).

In addition, PKC belongs to the serine/threonine protein kinase family that regulates the function of other proteins through the phosphorylation of these proteins. It has been demonstrated that PKC is directly involved in multiple steps of the TLR4-mediated signalling pathways, and the inhibition of PKC reduced cytokine expression in LPS-activated macrophages [29]. The levels of phosphorylated PKC in PPA-stimulated macrophages were analysed using Western blotting to determine the potential involvement of PKC in TNF-α and IL-6 expression. We demonstrated that 10 μg/mL of PPA increased the phosphorylation levels of PKC-α (*p* < 0.001) and PKC-δ (*p* < 0.05 at 10 min; *p* < 0.01 at 20–60 min) (Figure 5A). We knocked down PKC-α (*p* < 0.001) and PKC-δ (*p* < 0.001) using shRNA to elucidate their roles in TNF-α and IL-6 expression in PPA-treated cells (Figure 5B). Significantly lower levels of TNF-α (*p* < 0.001) (Figure 5C) and IL-6 (*p* < 0.001) (Figure 5D) were detected in PKC-α knockdown cells (sh-PKC-α) than those in mock control cells (sh-SC). Conversely, the PPA-induced level of secreted TNF-α in PKC-δ knockdown cells (sh-PKC-δ) was higher than that in sh-SC cells (*p* < 0.001) (Figure 5C). However, the expression level of IL-6 in PPA-activated sh-SC cells and sh-PKC-δ cells was not significantly different (Figure 5D).

### 3.4. The Role of Mitogen-Activated Protein Kinases (MAPK) and NF-Κb on TNF-A, IL-6 and COX-2 Expression in PPA-Activated Macrophages

**Figure 6:**
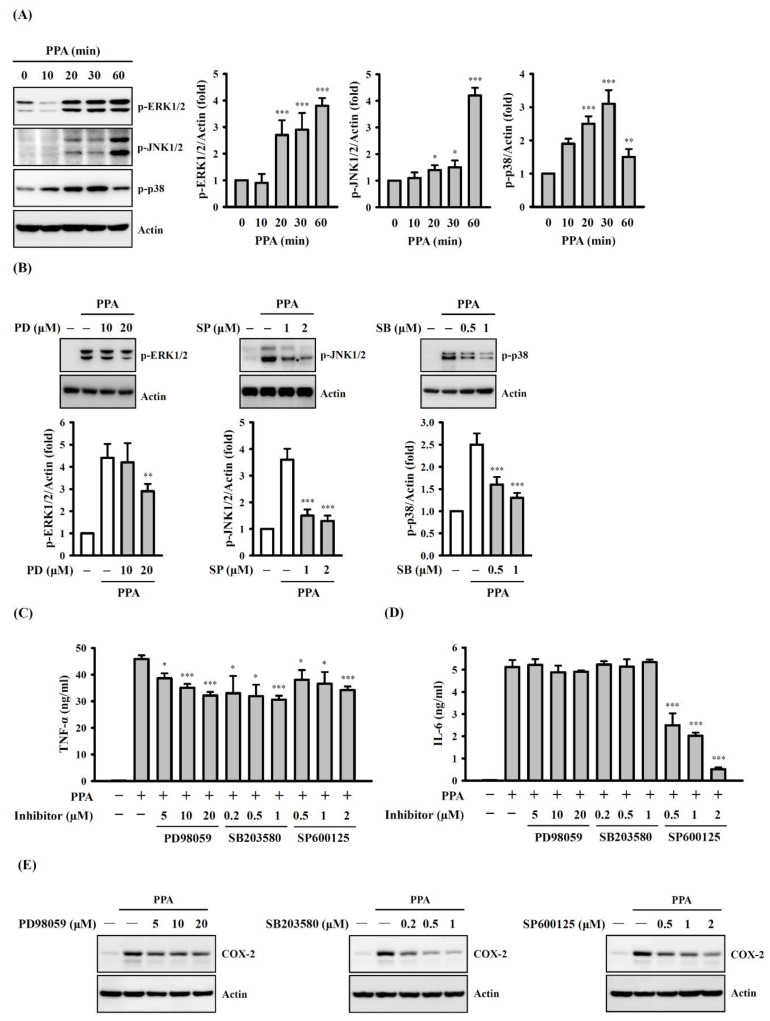
Effect of MAPK on PPA-induced TNF-α and IL-6 expression in RAW264.7 macrophages. (**A**) The phosphorylation levels of ERK1/2, JNK1/2 and p38 in PPA-stimulated cells. (**B**) Effect of MAPK inhibitors PD98059 (PD), SB203580 (SB) or SP600125 (SP) on the phosphorylation levels of ERK1/2, JNK1/2 and p38 in PPA-stimulated cells. (**C**–**E**) Effect of MAPK inhibitors on (**C**) TNF-α, (**D**) IL-6 and (**E**) COX-2 expression in PPA-stimulated cells. * *p* < 0.05, ** *p* < 0.01 and *** *p* < 0.001 compared to control cells (**A**) or PPA-treated cells (**B**–**D**).

MAPK are protein kinases that phosphorylate the serine and threonine residues of themselves or of their substrates to activate or de-activate their target. MAPK are composed of RK1/2, JNK1/2 and p38, which induce the expression of multiple genes that together regulate the inflammatory response [30]. Levels of MAPK phosphorylation in PPA-activated macrophages were analysed to determine whether MAPK participated in TNF-α and IL-6 expression. We demonstrated that 10 μg/mL of PPA significantly increased the phosphorylation levels of ERK1/2 (*p* < 0.001 at 20–60 min), JNK1/2 (*p* < 0.05 at 20 and 30 min; *p* < 0.001 at 60 min) and p38 (*p* < 0.001 at 20 and 30 min; *p* < 0.01 at 60 min) (Figure 6A), and these effects were reduced by the ERK1/2 inhibitor PD98059 (*p* < 0.05 at 5 μM), JNK1/2 inhibitor SP600125 (*p* < 0.001) and p38 inhibitor SB203580 (*p* < 0.001), respectively (Figure 6B). In addition, TNF-α expression, induced by 10 μg/mL of PPA, was reduced by PD98059 (*p* < 0.05 at 5 μM; *p* < 0.001 at 10 and 20 μM), SP600125 (*p* < 0.05 at 0.2 and 0.5 μM; *p* < 0.001 at 1 μM) and SB203580 (*p* < 0.05 at 0.5 and 1 μM; *p* < 0.001 at 2 μM) (Figure 6C), but PPA-induced IL-6 secretion was only reduced by SP600125 (*p* < 0.001) (Figure 6D). Thus, PPA induces TNF-α secretion through ERK1/2, JNK1/2, and p38, while PPA induces IL-6 secretion through JNK1/2. Furthermore, COX-2 expression, induced by 10 μg/mL of PPA, was reduced by PD98059, SP600125 and SB203580 (Figure 6E), indicating that PPA induces COX-2 expression through MAPK.

**Figure 7:**
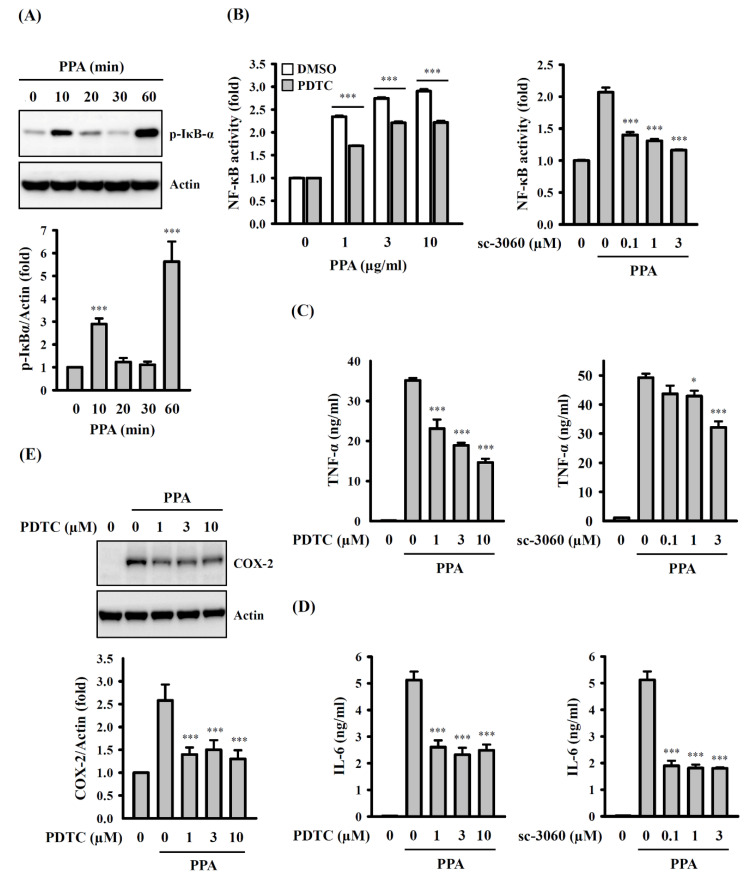
Effect of NF-κB on PPA-induced TNF-α and IL-6 secretion and COX-2 expression. (**A**) The phosphorylation levels of IκB-α in PPA-stimulated RAW264.7 macrophages. (**B**) Effect of PDTC or sc-3060 on NF-κB activation in PPA-stimulated RAW-Blue cells. (**C**–**E**) Effect of PDTC or sc-3060 on (**C**) TNF-α, (**D**) IL-6 and (**E**) COX-2 in PPA-stimulated RAW264.7 macrophages. * *p* < 0.05 and *** *p* < 0.001 compared to control cells (**A**), PPA-treated cells (**B**–**E**), or as indicated ((**B**), left panel).

Furthermore, a proinflammatory mediator was also regulated by transcription factor NF-κB. Our results showed an increase in the level of phosphorylated IκB-α at 10 min after 10 μg/mL of PPA treatment (*p* < 0.001), a decrease after 20 min, and an increase again at 60 min (*p* < 0.001) (Figure 7A). In addition, we demonstrated that PPA increased NF-κB transcriptional activity, and this effect was inhibited by the NF-κB inhibitors PDTC, a metal-chelating compound that potently and reversibly inhibits NF-κB (*p* < 0.001) (Figure 7B); however, PDTC also exerts antioxidant properties. Therefore, to confirm the role of NF-κB on inflammatory mediator expression in PPA-stimulated macrophages, another NF-κB inhibitor, sc-3060, a cell-permeable inhibitory peptide that inhibits the translocation of the NF-κB active complex into the nucleus, was used. We found that sc-3060 also significantly inhibited the NF-κB activation that was mediated by 10 μg/mL of PPA (*p* < 0.001) (Figure 7B). In addition, TNF-α (Figure 7C) and IL-6 (Figure 7D) expression, induced by 10 μg/mL of PPA, was reduced by PDTC (*p* < 0.001) and sc-3060 (for TNF-α, *p* < 0.05 at 1 μM, *p* < 0.001 at 3 μM; for IL-6, *p* < 0.001). PDTC also significantly reduced COX-2 expression in 10 μg/mL of PPA-activated macrophages (*p* < 0.001) (Figure 7E). Taken together, these results indicated that NF-κB positively regulated TNF-α, IL-6 and COX-2 expression in PPA-stimulated macrophages.

### 3.5. ROS, MAPK, and PKC Function Upstream of NF-κB in PPA-Activated Macrophages

**Figure 8:**
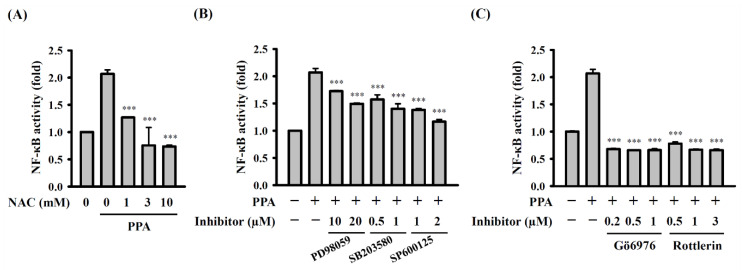
ROS, MAPK and PKC-α/δ function upstream of NF-κB in PPA-activated macrophages. Effect of (**A**) NAC, (**B**) MAPK inhibitors or (**C**) PKC inhibitors on NF-κB activation in PPA-stimulated RAW-Blue cells. *** *p* < 0.001 compared to PPA-treated cells.

We further investigated the crosstalk of intracellular signalling pathways in PPA-activated RAW264.7 macrophages. ROS acted upstream of NF-κB, as NF-κB activation, mediated by 10 μg/mL of PPA, was significantly inhibited by NAC (*p* < 0.001) (Figure 8A). In addition, NF-κB activation, mediated by 10 μg/mL of PPA, was reduced by MAPK inhibitors (*p* < 0.001) (Figure 8B). These results indicated that PPA induced NF-κB activation partially through MAPK. Furthermore, PKC-α and PKC-δ also played important roles in NF-κB activation in the RAW264.7 macrophages that were activated by 10 μg/mL of PPA, because the PKC-α inhibitor Gö6976 (*p* < 0.001) and PKC-δ inhibitor rottlerin (*p* < 0.001) significantly suppressed NF-κB activation (Figure 8C).

### 3.6. PPA Reduces the Phagocytic Activity and Cytokine Production of Macrophages in Response to Bacterial Infection

**Figure 9:**
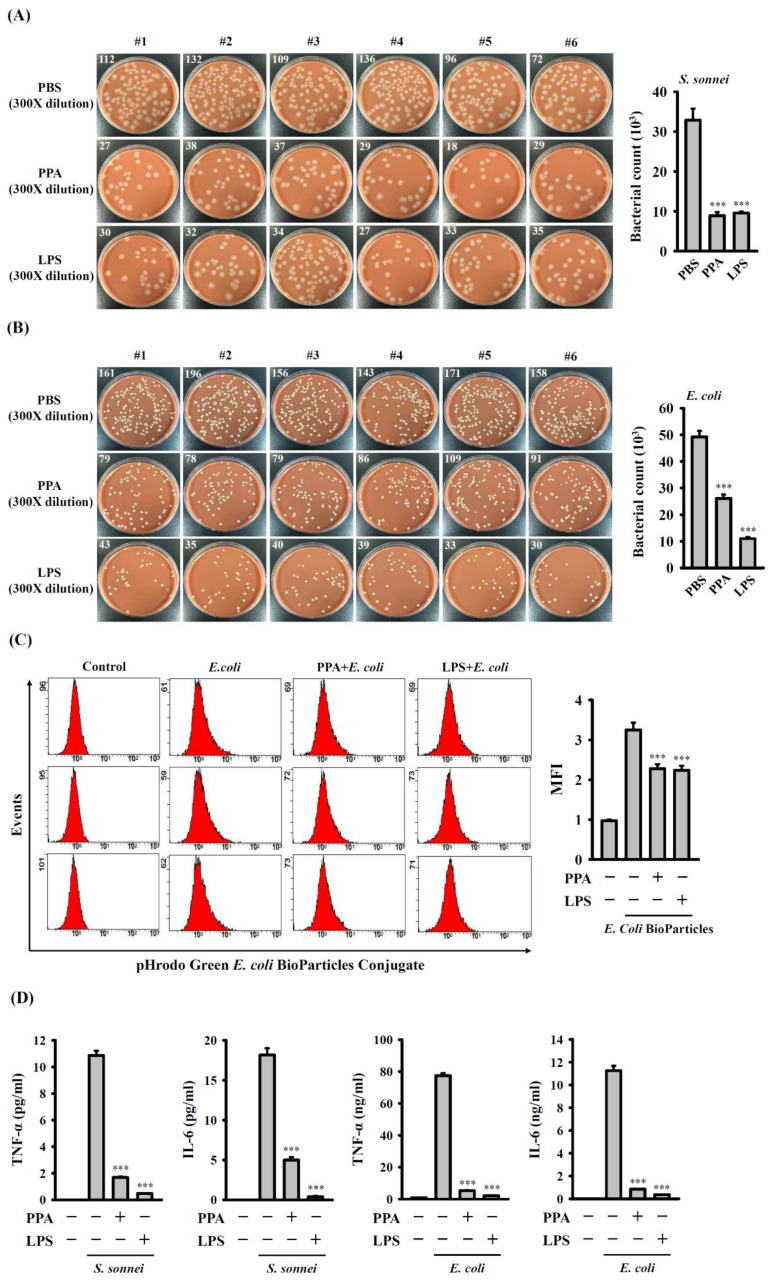
PPA reduces the phagocytic activity and cytokine production of J774A.1 macrophages in response to bacterial infection. (**A**–**C**) Effect of PPA on the phagocytic activity of cells in response to (**A**) *S. sonnei* infection, (**B**) *E. coli* infection or (**C**) pHrodo Green *E. coli* BioParticles Conjugat treatment. (**D**) Effect of PPA on the TNF-α and IL-6 production of cells in response to *S. sonnei* or *E. coli* infection. *** *p* < 0.001 compared to PBS-treated cells (**A**,**B**), *E. coli* BioParticles-treated cells (**C**) or bacteria-infected cells (**D**).

The effect of PPA on the phagocytosis activity of macrophages was investigated. Macrophages were treated with 10 µg/mL of PPA, 0.1 µg/mL of LPS or 5 µL/mL of PBS for 24 h, followed by infection with *S. sonnei* for 15 min. The number of engulfed *S. sonnei* in macrophages was analysed using the CFU assay. We observed a greater number of engulfed *S. sonnei* in PBS-treated cells (32,850 ± 2900 CFUs) than in PPA-treated cells (8900 ± 895 CFUs) (*p* < 0.001) or LPS-treated cells (9550 ± 358 CFUs) (*p* < 0.001) (Figure 9A). Under the same conditions, the number of engulfed *E. coli* in PPA-treated cells (26,100 ± 1459 CFUs) (*p* < 0.001) or LPS-treated cells (11,000 ± 593 CFUs) (*p* < 0.001) was lower than that in PBS-treated cells (49,250 ± 2206 CFUs) (Figure 9B). The phagocytosis of fluorescent *E. coli* particles by macrophages was analysed to confirm the effect of PPA on the phagocytic activity of macrophages. The intracellular fluorescence in 10 µg/mL of PPA-treated cells (*p* < 0.001) or 0.1 µg/mL of LPS-treated cells (*p* < 0.001) was lower than that in PBS-treated cells (Figure 9C). These results indicate that PPA treatment decreased the phagocytic activity of the macrophages. In addition, for 5 h *S. sonnei*- or *E. coli*-infected macrophages, being incubated with 10 µg/mL of PPA or 0.1 µg/mL of LPS for 24 h exhibited significantly reduced TNF-α (*p* < 0.001) and IL-6 (*p* < 0.001) expression (Figure 9D).

## 4. Discussion

It is necessary to check for possible LPS contamination in the samples when investigating their immune modulatory properties, as macrophages are highly sensitive to LPS [28]. Although we prepared PPA carefully to reduce any possible LPS contamination, an LAL test and PMB were used to exclude the possibility of LPS contamination. In addition, PPA did not induce iNOS expression or NO production in the macrophages, while LPS significantly increased their levels. The level of IL-1β production in PPA-treated cells was significantly lower than that in the LPS-treated cells. Furthermore, the inhibition of MAPK using pharmaceutical inhibitors significantly reduced TNF-α production in the LPS-activated macrophages [31]; however, PPA-mediated TNF-α production was only slightly reduced by these inhibitors. The inhibition of ERK1/2 with PD98059 significantly inhibited LPS- but not PPA-induced IL-6 production in macrophages [32]. Taken together, we demonstrated that the PPA activated the macrophages, and that this was not due to LPS contamination.

The activation of TLR4 by LPS induces MyD88-dependent and MyD88-independent pathways, the former controlling the expression of proinflammatory cytokine and the latter controlling Type I interferons and interferon-inducible genes [33]. In the MyD88-dependent pathways, MyD88 recruits and activates a death domain-containing kinase, IL-1 receptor-associated kinase-4 (IRAK-4), followed by the recruitment and activation of IRAK-1 [34]. In addition, LPS activates NF-κB and MAPK through TNF receptor-associated factor 6/transforming growth factor-β-activated kinase 1 [35]. In the MyD88-independent pathways, LPS activates NF-κB and MAPK through receptor-interacting protein 1, which is activated by a TRIF-related adaptor molecule/TIR domain-containing adaptor, inducing IFN-β [36,37]. In our previous study, we demonstrated that the activation of TLR4 by LPS induces ROS production through NADPH oxidase and increases the phosphorylation levels of MAPK and AKT and NF-κB activation in macrophages; notably, ROS positively regulated NF-κB activation, but did not regulate the phosphorylation levels of MAPK and AKT [38]. PPA and LPS regulate TNF-α and IL-6 production through similar, but not identical, pathways. It has been demonstrated that the activation of NF-κB is achieved through the phosphorylation-dependent degradation of IκB-α, which results in the release and subsequent nuclear translocation of NF-κB into the nucleus. In the previous report, Fujihara et al. showed that the almost complete degradation of IκB-α had occurred by 30 min after LPS treatment, with re-expression observed by 60 min [39]. The level of phosphorylated IκB-α peaked at 15 min after LPS treatment, decreased after 30 min, and increased again at 60 min [39]. These results were similar to our results of PPA-treated cells. We suggest that the reason why the phosphorylation level of IκB-α increased again at 60 min after PPA treatment may be due to the re-expression of the total IκB-α protein; however, the detailed mechanism needs further investigation. Although PPA activates macrophages through signalling pathways similarly to LPS, the role of TLR4 and MyD88 in PPA-mediated macrophage activation requires further investigation.

It has been demonstrated that ROS acted upstream of MAPK in LPS-activated J774A.1 macrophages [40]. Interestingly, our previous study demonstrated that a capsular polysaccharide isolated from *Klebsiella pneumonia* induced ROS production and MAPK phosphorylation through TLR4 in J774A.1 macrophages; however, the inhibition of ROS by NAC did not affect the phosphorylation levels of MAPK [41]. In addition, PKC-δ phosphorylation was inhibited by NAC, while PKC-α phosphorylation was increased by NAC in capsular polysaccharide-stimulated J774A.1 macrophages [41]. These results indicated that the relationship among ROS, MAPK and PKCs depended on the stimulators or cell types. In this study, we demonstrated that ROS, MAPK and PKCs function upstream of NF-κB in PPA-activated macrophages; however, the upstream and downstream relationship among ROS, PKCs and MAPK in PPA-activated macrophages needs further investigation. We also demonstrated that PPA induced TNF-α and IL-6 expression through ROS. Intracellular ROS can be generated from several enzymes or organelles, including NADPH oxidase, xanthine oxidase, cyclooxygenase, iNOS or mitochondria. In our previous study, we found that LPS induced ROS production through NADPH oxidase in J774A.1 macrophages [38]. It is important to dissect the detailed mechanism of ROS generated from PPA-activated macrophages in the future. Furthermore, ROS play important roles in signalling and in the pathogen killing in the phagocytes [42]. In this study we demonstrated that the ROS generated by PPA are required for signalling in the regulation of inflammatory mediator expression. However, whether the ROS generated by PPA contribute to the bactericidal activity of macrophages needs further investigation.

Polysaccharides have attracted significant attention from scientists due to their immunomodulatory properties. We isolated galactomannan with an octasaccharide-repeating unit from the Taiwanese medicinal fungus *Antrodia cinnamomea* [43] and glucuronoxylomannan from the wood ear mushroom *Auricularia auricula-judae* [44], and these two polysaccharides activate macrophages by inducing cytokine production. Furthermore, carboxylic and O-acetyl moieties are essential for the immunostimulatory activity of glucuronoxylomannan [44]. Toll-like receptors expressed on macrophages are important cellular receptors that recognize pathogen-associated molecular patterns and transduce signals leading to an inflammatory response. As shown in our previous studies, capsular polysaccharides from pathogenic *Klebsiella pneumonia* activate macrophages through TLR4 [28,39]. This is not limited to pathogenic polysaccharides, for increasing evidence has shown that polysaccharides from edible materials also activate macrophages through TLR4. We found that polysaccharides from *Antrodia cinnamomea* [45], *Auricularia auricula-judae* [44], and *Ganoderma lucidum* [46] activate macrophages through TLR4. Although PPA activates macrophages through signalling pathways similarly to LPS, the role of TLR4 in PPA-mediated macrophage activation requires further investigation.

Phagocytosis is an important innate immune defence process by which immune cells use their plasma membrane to engulf pathogens or cell debris. The first step in phagocytosis is the recognition of microbial pathogens by the cell surface receptors that bind pathogen-associated molecular patterns or opsonins [47]. The well-known pattern-recognition receptors are macrophage scavenger receptor class A, which binds LPS on Gram-negative bacteria, Dectin-1, which binds fungal beta-glucan, and mannose receptors that bind to mannan [47]. Phagocytosis not only destroys the ingested pathogens, but also induces an inflammatory response by activating intracellular signalling [46]. The inhibition of phagocytosis by the pharmaceutical inhibitors, cytochalasin D and colchicine, reduces LPS uptake and cytokine production in LPS-activated macrophages [46]. A previous study indicated that polysaccharides from *G. lucidum* inhibit phagocytic activity and reduce the expression of IL-1β and IL-6 in LPS- or amyloid-beta-activated microglia [48]. Fucoidan, a long-chain, sulfated polysaccharide from brown algae, reduces the phagocytosis of red blood cells by macrophages by blocking the macrophage scavenger receptor class A [49].

In general, immunomodulatory substances, which induce the secretion of proinflammatory cytokines, enhance phagocytosis. It has been demonstrated that phagocytosis activity was reduced in LPS-tolerant macrophages, but LPS-tolerant macrophages exhibited enhanced hydrogen peroxide production and antigen presentation molecule expression. These results suggested that LPS tolerance drives macrophages from a proinflammatory mediator-producing stage toward an enhanced antimicrobial activity state [50]. However, an opposite finding was reported by Pena et al., showing that the scavenger receptors MARCO and CD23, the key genes related to phagocytosis, were strongly upregulated during LPS tolerance compared with expression after a single LPS treatment [51]. In our previous study, we found that glucuronoxylomannan from the edible wood ear mushroom, *Auricularia auricula-judae,* induced LPS tolerance, but enhanced the phagocytosis of macrophages [44], which is consistent with the findings reported by Pena et al. These finding suggest that the phagocytosis activity of macrophages in LPS-tolerant macrophages may depend on different conditions. In the present study, a PPA treatment lasting 24 h reduced the phagocytosis of *S. sonnei* and *E. coli* by macrophages; however, further studies are needed to determine whether PPA inhibits phagocytosis by reducing the expression of pattern-recognition receptors or the key proteins related to phagocytosis.

In this study, we found that a PPA treatment for 24 h induced an LPS tolerance-like phenomenon in macrophages, as PPA reduced TNF-α and IL-6 expression in *S. sonnei*- or *E. coli*-infected macrophages. LPS tolerance can be induced by the down-regulation of the important TLR4-mediated signalling pathways, including MAPK phosphorylation and NF-κB activation [45]. We suggested that the reduced cytokine expression may be due to the suppressed phagocytosis of bacteria and the reduced expression of toll-like receptors or reduced MAPK phosphorylation and NF-κB activation. In addition, the gene profile responses of proinflammatory mediators in human peripheral blood mononuclear cells during endotoxin tolerance were similar to those observed during M2 polarization [51]. Notably, the PPA activated macrophages with an overexpression of cytokines in vitro, but inactivated the macrophages that were presented with a reduction of cytokine production while infected by bacteria. These results suggested that after an initial inflammatory stimulus, PPA-treated macrophages undergo an M2-like polarization, probably to control hyperinflammation. However, the effect of PPA on M1-M2 polarization of macrophages should be further investigated by analysing the M1/M2 macrophage markers.

Although PPA did not induce iNOS expression or NO production and produced lower levels of IL-1β than LPS in the macrophages, we did not test the effects of PPA on the activation of other immune cells, for example, neutrophils and mast cells. In addition, the main limitation of this study is that the structure–activity relationship of PPA has not yet been systematically elucidated. The sulfate groups of PPA may play important roles in its immune modulatory activities; however, this needs further investigation. Another limitation is the lack of an animal model to assess the immunomodulatory activity of PPA in vivo. Investigations of the effect of the administration of PPA to mice on cytokine expression and the immune response upon bacterial infection are important. Based on the current findings, we cannot conclude that PPA did not cause severe inflammation similarly to LPS. Taken together, this study provides scientific evidence for the immunomodulatory properties of polysaccharides from the coral *Pseudopterogorgia americana*, suggesting the potential of PPA to be an immunomodulatory in the future.

## 5. Conclusions

**Figure 10:**
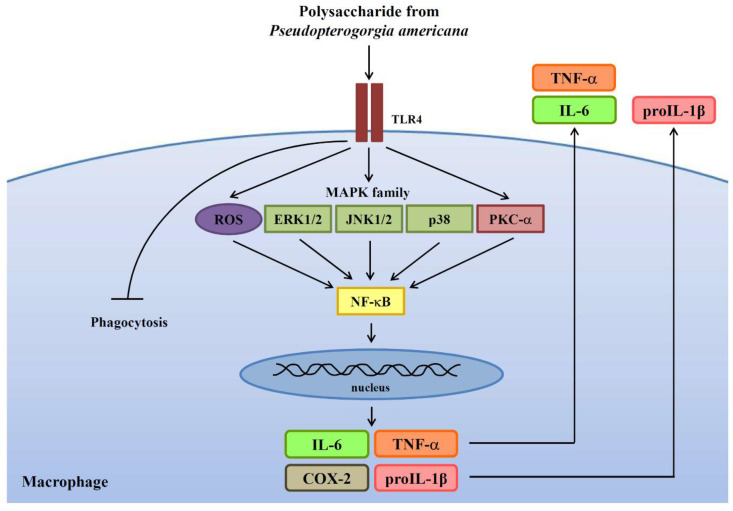
Overview of the putative mechanisms by which PPA activated macrophages.

The polysaccharides from the coral *Pseudopterogorgia americana* (PPA) activate macrophages through TLR4. PPA induces pro-inflammatory mediator expression in macrophages through ROS-, MAPK-, PKC-α/δ- and NF-κB-dependent pathways (Figure 10). PPA pre-treatment decreased the phagocytosis activity of macrophages and reduced cytokine expression in bacteria-infected macrophages.

## Data Availability

The datasets in this study are available from the corresponding author upon reasonable request.
